# Effects of FTO Gene rs9939609 and rs17817449 Polymorphisms on Insulin Resistance in Turkish Children With Obesity

**DOI:** 10.1111/1753-0407.70213

**Published:** 2026-03-20

**Authors:** Merve Köksal, Mehtap Ünlü Söğüt, Sevtap Kabalı

**Affiliations:** ^1^ Department of Obstetrics and Pediatrics Clinic Samsun Education and Research Hospital Samsun Türkiye; ^2^ Department of Nutrition and Dietetics Faculty of Health Sciences, Ondokuz Mayıs University Samsun Türkiye

**Keywords:** children, FTO gene, insulin resistance, rs17817449, rs9939609

## Abstract

**Backgrounds:**

Fat mass and obesity‐related gene (FTO) has been associated with glucose homeostasis and insulin sensitivity in some populations. The aim of this study was to evaluate the association of FTO gene rs9939609 and rs17817449 polymorphisms with insulin resistance, some cardiometabolic parameters, and body mass index (BMI) in Turkish children with obesity.

**Methods:**

The cross‐sectional observational study included 83 children with obesity genotyped for variants of the FTO gene (rs9939609 and rs17817449). Children were divided into two groups as control and insulin resistant according to the homeostatic model assessment of insulin resistance (HOMA‐IR). The relationship between the genotypes of rs9939609 and rs17817449 polymorphisms and insulin resistance was determined using binary logistic regression analysis.

**Results:**

In the control group and insulin‐resistant obese group, the majority of the rs9939609 polymorphism was AT (69.2%) and AA (64%) genotypes, and the majority of the rs17817449 polymorphism was GT (65.4%) and TT (70%) genotypes, respectively. Fasting glucose and insulin values were lower, and quantitative insulin sensitivity calculation index (QUICKI) values were higher in the risk genotype (GG) of rs17817449 polymorphism (*p* < 0.05). Additionally, rs9939609 (AT genotype, OR = 0.063; 95% CI: 0.006–0.707, *p* = 0.025 and AA genotype, OR = 0.111; 95% CI: 0.021–0.576, *p* = 0.009) and rs17817449 (GT genotype, OR = 12.250; CI: 9.720–84.383, *p* < 0.001) polymorphisms were strongly associated with insulin resistance.

**Conclusion:**

Our study supports that FTO gene rs9939609 and rs17817449 polymorphisms are associated with insulin resistance independently of age, gender, and BMI.

## Highlights

- In children with insulin resistance, the majority of the rs9939609 polymorphism was AA and the majority of the rs17817449 polymorphism was TT genotypes.
- A relationship was found between the GT genotype of the rs17817449 polymorphism of the FTO gene and insulin resistance.
- The AA and AT genotypes of the rs9939609 polymorphism of the FTO gene were determined to be associated with insulin resistance.
- The QUICKI value, which indicates insulin sensitivity, showed differences among the genotypes of the rs17817449 polymorphism of the FTO gene.

## Graphical abstract

Impact of FTO polymorphisms (rs9939609 and rs17817449) on glycemic traits, insulin homeostasis, and metabolic indicators.

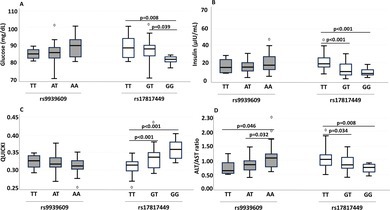

## 1 Introduction

Obesity is a disease resulting from excessive accumulation of fat in the body [1]. The prevalence of childhood obesity was found to have doubled in many countries between 1990 and 2022 [2]. It is known that genetic and many environmental factors are effective in the etiology of obesity [1]. Excessive and unhealthy nutrition, insufficient physical activity, increased time spent in front of the screen (TV, computer, tablet, phone, etc.) can be given as examples of environmental factors [3]. However, genetic susceptibility is an important factor among the causes of obesity. Many studies have suggested that the fat mass and obesity‐related gene (FTO) plays important roles in the development of obesity [4, 5, 6].

The human FTO gene is a large nucleic acid demethylase spanning nine exons, extending over 400 kb on chromosome 16q12.2 [7]. Some studies in the literature have highlighted the association of the FTO gene, especially rs9939609 and rs17817449 single nucleotide polymorphisms (SNPs) with childhood obesity [8, 9]. FTO gene rs9939609 polymorphism has A and T alleles [10], while rs17817449 polymorphism has T and G alleles [11]. In genome studies, the association between the A allele of the rs9939609 polymorphism and the G allele of the rs17817449 polymorphism with obesity phenotype is frequently analyzed [11, 12]. Frayling et al. found that some SNPs belonging to the FTO gene were associated with body mass index (BMI) in the “Genome‐Wide Association Study (GWAS)” comparing 2938 healthy individuals with 1924 Type 2 diabetic patients in Europe [4]. At the same time, FTO gene polymorphisms have been found to increase body fat and metabolic syndrome risk independent of ethnicity, gender, and age [13].

Insulin resistance is when the pancreas secretes more insulin than normal to compensate for reduced insulin sensitivity in insulin‐targeted muscle, liver, and fat tissues. In cases where the pancreas cannot meet the high insulin demand and glucose input to the tissues is insufficient, Type 2 diabetes may occur with an increase in blood glucose levels [14]. Genetic studies have focused on gene expressions [15] and polymorphisms [16] that are effective in the emergence of insulin resistance.

The association of the FTO gene with insulin resistance and Type 2 diabetes is a subject of current research [16]. Silencing of FTO expression has been reported to inhibit insulin secretion by affecting metabolic signaling [17]. In addition, it has been suggested that the FTO gene may be effective in the function of beta cells [18], glucose homeostasis, and regulation of insulin sensitivity [19]. However, there is a limited number of studies investigating the relationship between FTO gene polymorphisms and insulin metabolism in childhood obesity. In addition, the lack of a study investigating the relationship between FTO gene polymorphisms and insulin resistance in Turkish children with obesity is one of the deficiencies in the literature. In this study, FTO gene rs9939609 and rs17817449 polymorphisms were analyzed in Turkish children with obesity and the association of polymorphisms with insulin resistance, some cardiometabolic parameters and BMI was evaluated.

## 2 Methods

### 2.1 Study Design and Population

**FIGURE 1:**
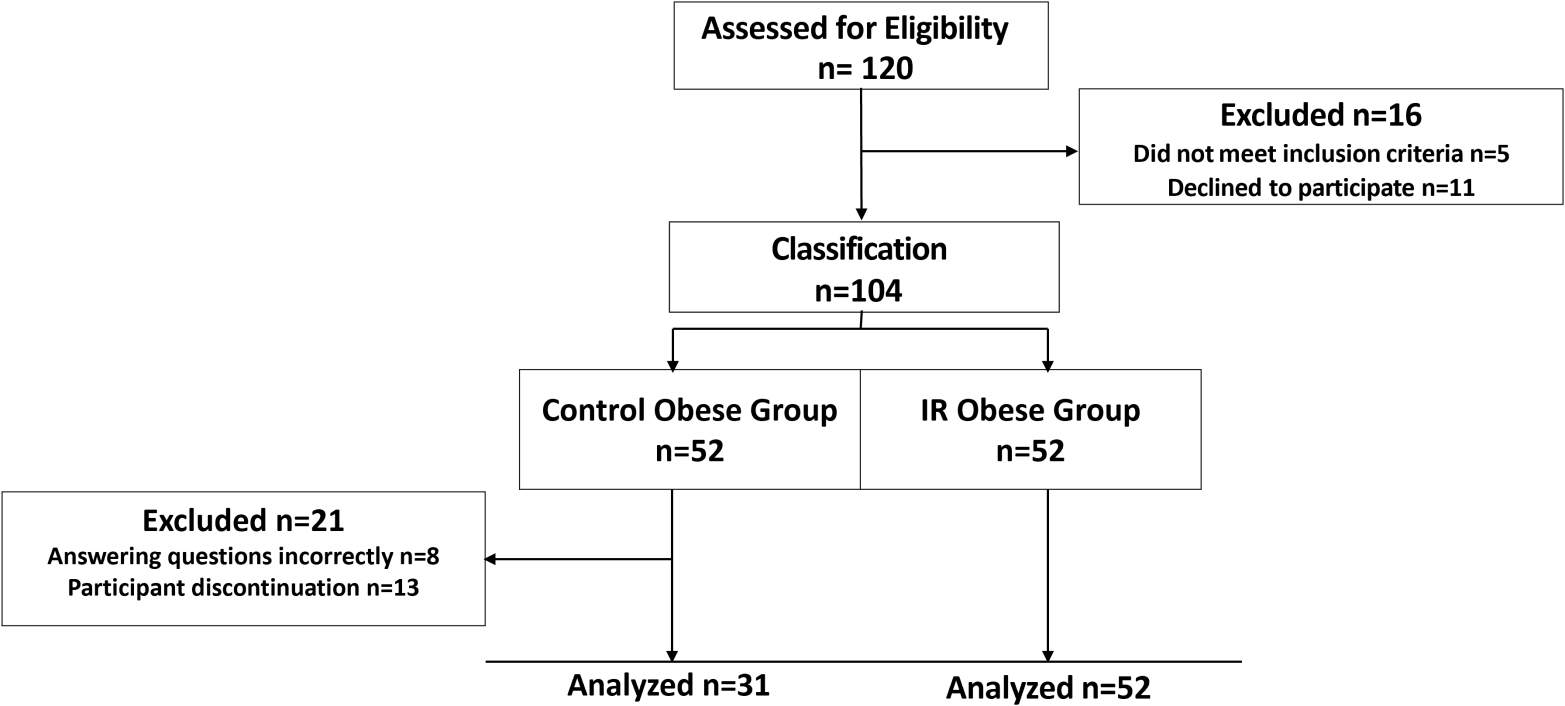
Study design. IR: insulin resistance.

This cross‐sectional observational study was conducted on 83 children aged 8–11 years who were treated at the Samsun Education and Research Hospital, Department of Obstetrics and Pediatrics Clinic. The necessary permissions were obtained from the Ministry of Health of the Republic of Türkiye for the study (Decision no. TUEK1‐2019BADK/1‐4, Date: 08.01.2019). The study was approved by the Clinical Research Ethics Committee, Ondokuz Mayıs University, Samsun, Türkiye (Decision no. 2018/269, Date: 25.06.2018) and all procedures were performed in accordance with the principles of the Declaration of Helsinki. Children with any chronic disease, using glucose‐lowering medication, and whose informed consent form was not signed by their parents were not included in the study. The parents and children were informed by the researchers about the purpose of the study and written informed consent was obtained from each of them. The study design is shown in Figure 1.

### 2.2 Sample Size

The sample size calculation for the study was performed using the G*power program (Version 3.1, Düsseldorf, Germany) and based on the study by Abdelmajed et al. [20]. The power analysis concluded that including 82 children in the study would be sufficient with 95% reliability and 5% sampling error. Although the goal was to have an equal number of children in both groups, there were dropouts from the study for various reasons, as shown in Figure 1. This led to changes in the study's actual power value and the number of participants per group. The study's actual power is 0.9497864, and the allocation ratio N2/N1 is 1.67.

### 2.3 Data Collection

Demographic characteristics, lifestyle habits, and health history of the children were evaluated using a face‐to‐face questionnaire.

### 2.4 Anthropometric Measurements

Anthropometric measurements of the children were made in accordance with the standardized anthropometric measurement guide with light clothing and without shoes [21]. Body weight was measured using the Tanita scale (Tanita bc 454 N Inner Scan, Japan) and height was measured using a stadiometer (Inbody BSM170, USA) with an accuracy of 0.1 cm. BMI = body weight (kg)/square of height (m^2^) was calculated by using these data. BMI by age was calculated using the WHO AnthroPlus program (Geneva, Switzerland). Accordingly, children with a *z*‐score ≥ 2 SD were considered obesity and included in the study [22].

### 2.5 Biochemical Measurements

The biochemical results of the patients were obtained retrospectively from the laboratory recording system of the hospital. Fasting glucose, fasting insulin, aspartate aminotransferase (AST), alanine aminotransferase (ALT), triglyceride, total cholesterol, HDL‐cholesterol (HDL‐C), LDL‐cholesterol (LDL‐C), and low‐density lipoprotein (VLDL) levels were recorded after an overnight fast (8–10 h) following dinner. The formula HOMA IR = [fasting glucose (mmol/L) × fasting insulin (mU/L)/22.5] was used to estimate insulin resistance. Patients with HOMA IR > 2.5 were considered to have insulin resistance [23]. Children were divided into two groups according to the presence of insulin resistance. The control obese group consisted of children with a HOMA‐IR value of 2.5 and below, and the insulin‐resistant obese (IR obese) group consisted of children with a HOMA‐IR value above 2.5. The control obese group was selected from obese children without IR rather than from healthy, normal‐weight children. The rationale for this choice was to control for the confounding effects of obesity itself and to specifically assess the independent impact of insulin resistance. In this way, obesity‐related influences were present in both groups, while the additional contribution of insulin resistance could be more clearly demonstrated. The cutoff value of HOMA‐IR > 2.5 to define insulin resistance has been widely used in pediatric populations and is supported by previous studies [24]. Therefore, we adopted the same criterion in our study. Additionally, the QUICKI formula = [1/(log fasting insulin + log fasting glucose)] was used to estimate insulin sensitivity.

### 2.6 DNA Extraction

For DNA extraction, 4 mL of peripheral venous blood was collected from each participant in an ethylenediamine tetra acetic acid (EDTA) tube. Blood samples were stored at −80°C until analysis. DNA extraction was performed using the PureLink Genomic DNA Mini Kit (Invitrogen, USA). Subsequently, the concentration and purity of isolated DNA collected in a microcentrifuge tube were measured using a NanoDrop UV spectrophotometer (Thermo Fisher Scientific, USA).

### 2.7 Genotyping

Real‐time polymerase chain reaction (PCR—T100, Bio‐Rad, Hercules, CA, USA) with hydrolysis‐based probe was used for the detection of intronic FTO gene rs9939609 (T/A) and rs17817449 (G/T) SNPs. In the genetic analysis for the FTO gene, rs9939609 was pre‐denaturation at 95°C for 4 min (first denaturation), denaturation at 94°C for 30 s (DNA chain opening), annealing at 58°C for 30 s (attachment/bonding of the primers to the opened DNA chain), primer extension at 72°C for 1 min, and final extension at 72°C for 10 min. Similarly, in the genetic analysis for the FTO gene rs17817449, the PCR protocol was as follows: pre‐denaturation at 94°C for 5 min, denaturation at 94°C for 30 s, annealing at 57°C for 30 s, primer extension at 72°C for 30 s 35 cycles, and final extension at 72°C for 5 min.

Restriction fragment length polymorphism analysis was performed to identify alleles of FTO gene rs9939609 and rs17817449 polymorphisms. Amplicons obtained for FTO gene rs9939609 polymorphism were incubated with 2U ScaI restriction enzyme for 6 h at 37°C. Based on FTO gene rs9939609 polymorphisms, the patients were divided into three groups according to having AA, AT, TT genotypes. In this context, AA genotype was considered as “homozygote genotype,” AT genotype as “heterozygote genotype” and TT genotype as “wild type genotype” in terms of risk allele. Likewise, the amplicons obtained for the FTO gene rs17817449 polymorphism were incubated with 2U 23 AlwN I enzyme for 6 h at 37°C. Based on FTO gene rs17817449 polymorphisms, the patients were divided into three groups according to their having TT, TG, GG genotypes. In this context, TT genotype was considered as “homozygote genotype,” TG genotype as “heterozygote genotype” and GG genotype as “wild type genotype” in terms of risk allele.

#### 2.7.1 Genotyping Quality Control

The purity and integrity of DNA samples were measured using a NanoDrop UV spectrophotometer (Thermo Fisher Scientific, USA), and samples with an A260/280 ratio < 1.8 were excluded from analysis. Genotyping calls (SNP rs9939609 and rs17817449) were obtained using automated software, and samples with low quality were re‐evaluated. A randomly selected 10% of samples were re‐genotyped, and the results showed 100% consistency. Genotype calls were independently verified by two researchers. The missing data rate per SNP was found to be < 5%, which was considered acceptable for the analyses. The groups' compliance with the Hardy–Weinberg equilibrium (HWE) test was checked and found to be compatible.

### 2.8 Statistical Analysis

The data obtained from the study were analyzed using SPSS software (Version 27.0 Inc., Chicago, IL, USA). The normality of the variables was evaluated by applying the Kolmogorov–Smirnov test. Categorical variables were expressed as frequency (percentage) and continuous variables were expressed as median and interquartile range (IQR 25th–75th percentile). Chi‐square test was used to compare gender, alleles, and genotypes according to the presence of insulin resistance, and Mann–Whitney *U* test was used to compare biochemical parameters. Fasting glucose, fasting insulin, QUICKI, and ALT/AST ratio according to FTO genotypes were evaluated by Kruskal–Wallis test. Binary Logistic Regression was performed for insulin resistance according to the genotypes of the children, and odds ratio (OR) and 95% confidence intervals (CI) were calculated. Two different regression models were created without adjustment and with adjustment for age, gender, and BMI. Statistically, *p* < 0.05 was accepted as significant.

## 3 Results

The study included 35 boys and 48 girls with a median age of 9 years (IQR 8.0–11.0). The median of body weight and height were found to be higher in the insulin‐resistant obese group (*p* < 0.05). According to BMI (*z* score) values, all of the children were found to be obese. Biochemical results of the children were within the normal range and the median values of fasting glucose [88.6 (IQR 83.0–93.6)], insulin [19.0 (IQR 15.6–25.7)], ALT [23.3 (IQR 15.7–32.6)] and ALT/AST ratio [0.9 (IQR 0.72–1.21)] were found higher in the insulin‐resistant obese group (*p* < 0.05) (Table 1).

**TABLE 1:** Descriptive characteristics and biochemical parameters of children.

Variables	Control obese (*n*: 31)	IR obese (*n*: 52)	Total (*n*: 83)	Reference	Statistics	*p*
	Median or *n* (IQR 25–75 or %)	Median or *n* (IQR 25–75 or %)	Median or *n* (IQR 25–75 or %)			
Sex						
Boy	12 (38.7)	23 (44.2)	35 (42.2)	—	0.243^a^	0.622
Girl	19 (61.3)	29 (55.8)	48 (57.8)	—		
Age (years)	9 (8–10)	10 (8.2–11.7)	9 (8–11)	—	−1.746^b^	0.081
Anthropometric measurements						
Weight (kg)	48 (42–55)	59.5 (51–67.4)	54.8 (46.9–65)	—	−3.516^b^	< 0.001**
Height (cm)	139.4 (136.9–146.5)	146.0 (139–153.7)	144.7 (138–152.5)	—	−2.133^b^	0.033*
BMI‐for‐age *z*‐score	2.8 (2.4–3.3)	2.9 (2.7–3.8)	2.9 (2.6–3.6)	—	−1.906^b^	0.057
Obese (> +2SD)	31 (100)	52 (100)	83 (100)		3.438^a^	0.064
Metabolic indicators						
Glucose (mg/dL)	84.2 (82–88.3)	88.6 (83–93.6)	86.7 (82.8–91.8)	60–100	−2.518	0.012*
Insulin (μIU/mL)	8 (5.3–9.6)	19 (15.6–25.7)	15.3 (9–23.5)	2.6–24.9	−7.517	< 0.001**
AST (U/L)	23.3 (21.3–28.3)	24.7 (20.4–28.3)	24.4 (20.7–28.3)	5–50	−0.122^b^	0.903
ALT (U/L)	17 (14.5–25.2)	23.3 (15.7–32.6)	20.4 (15.2–29.5)	5–50	−2.010^b^	0.044*
ALT‐to‐AST‐ratio	0.7 (0.61–0.89)	0.9 (0.72–1.21)	0.8 (0.70–1.12)	—	−2.881^b^	0.004*
Triglycerides (mg/dL)	95 (59–120)	95 (74.5–121.7)	95 (73–120)	0–150	−1.681^b^	0.093
Total cholesterol (mg/dL)	163 (149–181)	162 (147–189)	162.5 (147–188)	0–200	−0.254^b^	0.799
HDL‐C (mg/dL)	45 (42–55)	46 (38.7–53.7)	45.5 (41–55)	40–60	−1.098^b^	0.272
LDL‐C (mg/dL)	93 (81–112)	97 (80.7–114.7)	96.5 (81–144)	0–130	−0.292^b^	0.770
VLDL (mg/dL)	17.8 (11.8–23.8)	19 (14.6–23.5)	18.9 (13.4–23.6)	10–40	−1.714^b^	0.087

Abbreviations: ALT, alanine transaminase; AST, aspartate transaminase; BMI, body mass index (*z*‐score); HDL‐C, high density lipoprotein cholesterol; IR obese, insulin‐resistant obese group; IQR, interquartile range; LDL‐C, low density lipoprotein cholesterol; *N*, number of participants; VLDL, very low density lipoprotein.

^a^ Chi‐square test.

^b^ Mann–Whitney *U* test.

*p < 0.05; **p < 0.001.

When the frequency of the genotypes of the FTO gene rs9939609 polymorphism in children was evaluated, it was found that 6.3% was TT, 39.7% was AT, and 54% was AA, while the genotypes belonging to the FTO gene rs17817449 polymorphism were 48.7% TT, 40.8% GT, and 10.5% GG (Table 2). In the control group and the insulin‐resistant obese group, the majority of the FTO gene rs9939609 polymorphism was in the AT (69.2%) and AA (64%) genotypes, respectively. The difference between the frequencies of the genotypes of the FTO gene rs9939609 polymorphism according to insulin resistance was statistically significant (*p* = 0.006). In addition, the majority of the FTO gene rs17817449 polymorphism in the control group and the insulin‐resistant obese group were GT (65.4%) and TT (70%) genotypes. The difference between the genotypes of the FTO gene rs17817449 polymorphism according to insulin resistance was statistically significant (*p* < 0.001).

**TABLE 2:** Genotype and allele frequencies of FTO genes according to insulin resistance.

Variables	Control obese	IR obese	Total	*χ* ^2^	*p*
	*n* (%)	*n* (%)	*n* (%)		
rs9939609					
Allels					
A	12 (54.5)	46 (69.7)	58 (65.9)	6.290	0.043*
T	10 (45.5)	20 (30.3)	30 (34.1)		
Genotypes					
TT	2 (15.4)	2 (4)	4 (6.3)	10.228	0.006*
AT	9 (69.2)	16 (32)	25 (39.7)		
AA	2 (15.4)	32 (64)	34 (54)		
rs17817449					
Allels					
G	24 (55.8)	15 (23.4)	39 (36.4)	30.300	< 0.001**
T	19 (44.2)	49 (76.6)	68 (63.6)		
Genotypes					
TT	2 (7.7)	35 (70)	37 (48.7)	34.205	< 0.001**
GT	17 (65.4)	14 (28)	31 (40.8)		
GG	7 (26.9)	1 (2)	8 (10.5)		

*Note:* IR obese, obese group with insulin resistance; genotypes depicted as count (%); Chi‐square test.

*p < 0.05; **p < 0.001.

**FIGURE 2:**
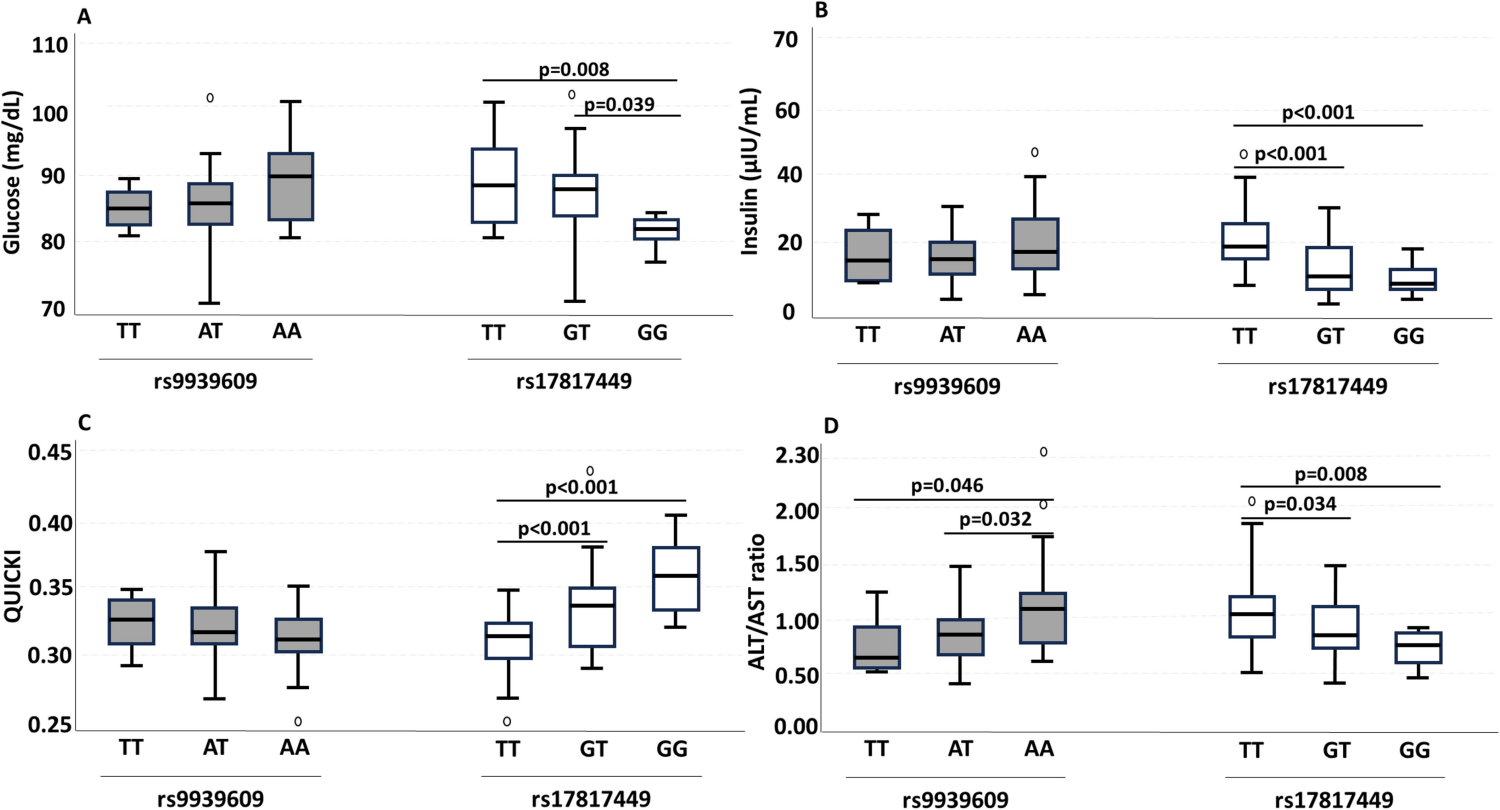
Evaluation of biochemical parameters according to FTO genotypes. Glucose (A), insulin (B), QUICKI (C), ALT/AST ratio (D); Kruskal–Wallis test was used for statistical analysis.

The relationships between some metabolic parameters and genotypes of FTO gene are shown in Figure 2. No significant difference was found between the genotypes of FTO rs9939609 gene in terms of fasting glucose, insulin and QUICKI values indicating insulin sensitivity (Figure 2A–C, *p* > 0.05). However, the median ALT/AST ratio of children with AA genotype was higher than that of children with TT and AT genotypes (Figure 2D, *p* < 0.05). In relation to the genotypes of the FTO rs17817449 gene, the glucose value of TT and GT genotyped children was higher than that of GG genotyped children (Figure 2A, *p* < 0.05). However, while the QUICKI value was lower in children with TT genotype than in children with GT and GG genotypes (Figure 2C, *p* < 0.001), insulin (Figure 2B, *p* < 0.001) and ALT/AST ratio (Figure 2D, *p* < 0.05) were found to be higher.

The relationship between insulin resistance and the FTO rs9939609 and rs17817449 genotypes were examined using a binary logistic regression model (Table 3). For the FTO gene rs9939609 polymorphism, the probability of insulin resistance was higher in children with AT and AA genotypes (AT genotype, OR = 0.063; 95% CI: 0.006–0.707, *p* = 0.025 and AA genotype, OR = 0.111; 95% CI: 0.021–0.576, *p* = 0.009), and this finding for the AA genotype persisted in the adjusted model (AA genotype, OR = 0.145; 95% CI: 0.019–1.113, *p* = 0.046). For the rs17817449 polymorphism, the GT genotype was significantly associated with an increased probability of insulin resistance in both unadjusted (OR = 12.250; 95% CI: 9.720–84.383, *p* < 0.001) and adjusted models (OR = 11.593; 95% CI: 0.737–82.360, *p* < 0.001) with an increased probability of insulin resistance. However, due to the wide CI, this result should be interpreted with caution.

**TABLE 3:** Relationships between the presence of insulin resistance and FTO rs9939609 and rs17817449 genotypes in children.

Genotypes	Binary logistic regression							
	Insulin resistance							
	Unadjusted model^a^				Adjusted model^b^			
	*B*	S.E.	OR (95% CI)	*p*	*B*	S.E.	OR (95% CI)	*p*
rs9939609								
TT	Reference				Reference			
AT	2.773	1.237	0.063 (0.006–0.707)	0.025*	2.413	1.426	0.90 (0.05–1.465)	0.091
AA	2.197	0.840	0.111 (0.021–0.576)	0.009*	1.931	1.049	0.145 (0.019–1.113)	0.046*
rs17817449								
TT	Reference				Reference			
GT	4.808	1.293	12.250 (9.720–84.383)	< 0.001**	4.753	1.406	11.593 (0.737–82.360)	< 0.001**
GG	1.752	1.128	5.765 (0.631–5.262)	0.121	1.713	1.168	5.543 (0.562–14.646)	0.142

*Note:* Absence of insulin resistance was accepted as the reference. For both polymorphisms, the TT genotype is the reference category. *Β*: Regression coefficient. Results with wide confidence intervals (e.g., rs17817449 GT genotype) should be interpreted with caution due to limited statistical precision.

Abbreviations: BMI, body mass index; CI, confidence interval; OR, odds ratio; SE, standard error.

^a^ Unadjusted.

^b^ Adjusted for age, gender and BMI.

*p < 0.05, **p < 0.001.

## 4 Discussion

Obesity is an important risk factor for metabolic and cardiovascular diseases [25]. In a large‐scale GWAS, a strong association between childhood obesity and FTO gene polymorphisms was identified [26]. Furthermore, FTO gene polymorphisms (rs17817449) have been found to be effective on BMI, fasting insulin, tissue glucose tolerance, and insulin resistance. A meta‐analysis (36 studies, *n* = 198.502) revealed that FTO gene polymorphisms (rs9939609) are associated with obesity and increased liver enzymes, blood pressure, lipids, hemoglobin A1C, fasting glucose, and insulin [27]. Although the association of these polymorphisms with obesity has been investigated, there is a limited number of studies investigating their effects on insulin resistance and fasting glucose. In this study, we evaluated the association of FTO gene rs9939609 and rs17817449 polymorphisms with insulin resistance, some cardiometabolic parameters, and BMI in Turkish children.

Insulin resistance, which is associated with FTO gene polymorphisms, is among the common complications of obesity [28]. Vigna et al. reported that individuals with insulin resistance have higher BMI and cardiometabolic risk factors such as TG, TC, and LDL‐C [29]. In this study, although the median body weight of the insulin‐resistant obese group was higher, BMI did not differ between the groups (*p* > 0.05). In addition, TG, TC, HDL‐C, and LDL‐C levels of the children were within the normal range, and ALT level and ALT/AST ratio among liver enzymes were found to be higher in the insulin‐resistant obese group (*p* < 0.05). In a similar study conducted in children with obesity, a relationship was found between insulin resistance and ALT levels and it was determined that increased liver enzymes were effective on decreased insulin sensitivity.

Studies have reported that 50%–63% of the European population has at least one of the FTO gene risk alleles [4, 26]. In a study conducted on Turkish adults (*n* = 1967), the relationship between heart diseases and risk factors and FTO gene rs1421085 and rs9939609 polymorphisms was investigated. The risk allele frequencies of these polymorphisms were found to be 42% and 39.8%, respectively. In addition, when the FTO gene rs9939609 polymorphism was analyzed, TT, AT, and AA genotype frequencies were found to represent 36%, 48.3%, and 15.7% of the population, respectively. In Turkish adults, polymorphisms in the FTO gene were found to contribute independently to obesity in women and to metabolic syndrome and insulin resistance in men through interaction with BMI [30]. Although different FTO gene polymorphisms have been investigated in the literature for Turkish adults, there are a limited number of studies investigating the relationship between FTO gene polymorphisms and cardiometabolic parameters in childhood obesity. In a study investigating the relationship between cardiometabolic parameters and FTO gene rs9939609 polymorphism in Turkish children with obesity, it was found that AA, AT, and TT genotype frequencies represented 26%, 48%, and 26% of the population, respectively. Besides, no significant difference was found between the genotypes of the FTO gene rs9939609 polymorphism in terms of HOMA‐IR values in children [31]. In this study, it was found that TT, AT, and AA genotype frequencies of rs9939609 polymorphism represented 6.3%, 39.7%, and 54% of the population, respectively. These results indicate that the prevalence of the risk allele is higher in the children who participated in the study. The frequencies of TT, GT, and GG genotypes of the other rs17817449 polymorphism were found to represent 48.7%, 40.8%, and 10.5% of the population, respectively. In addition, the difference between the genotypes of rs9939609 (*p* = 0.006) and rs17817449 (*p* < 0.001) polymorphisms according to the presence of insulin resistance was statistically significant. Like this study, the effect of FTO gene rs9939609 and rs17817449 polymorphisms on obesity and cardiometabolic parameters was investigated in Egyptian children and adolescents. It was reported that AA genotype (71%) for rs9939609 polymorphism and GG genotype (44%) for rs17817449 polymorphism were more common in the population. At the same time, no association was found between polymorphisms and fasting glucose [20]. Turkish and Egyptian children were similar in terms of the prevalence of rs9939609 polymorphism and different in terms of rs17817449 polymorphism. These results support the view that current gene polymorphisms should be investigated in children with different ethnic origins of obesity.

In this study, fasting glucose, insulin, QUICKI value indicating insulin sensitivity, and ALT/AST ratios of genotypes of FTO gene rs9939609 and rs17817449 polymorphisms were compared. In accordance with previous studies [31, 32], no significant difference was found between genotypes of rs9939609 polymorphism in terms of fasting glucose, fasting insulin, and QUICKI values (*p* > 0.05). Nascimento et al. reported that QUICKI value differed between genotypes of rs9939609 polymorphism and insulin sensitivity was affected by exercise duration in children and adolescents [33]. Interestingly, children with the risk genotype (GG) of the rs17817449 polymorphism had lower fasting blood glucose and insulin levels, while the QUICKI value was higher compared to those with other genotypes (*p* < 0.05). Consistent with our findings, a study in the Polish population reported no association between Type 2 diabetes and the GG genotype [34]. In contrast, Barseem et al. reported that the risk allele (G) was associated with higher fasting glucose and lower QUICKI values in Egyptian children [35]. Similarly, other studies have shown a higher frequency of the G allele and the GG genotype in individuals with Type 2 diabetes [36, 37]. These conflicting results suggest that insulin sensitivity may vary depending on the type of polymorphism, sample size, ethnicity, lifestyle, and environmental factors. Therefore, to better understand the role of this variant in glucose metabolism, more studies with larger samples from different populations are needed.

As another important result of the study, after adjustment for age, sex, and BMI, the risk genotype (AA) of rs9939609 polymorphism was strongly associated with insulin resistance (OR = 0.145; 95% CI: 0.019–1.113, *p* = 0.046). In addition, the heterozygous genotype (GT) of rs17817449 polymorphism was found to be associated with insulin resistance (OR = 12.250; 95% CI: 9.720–84.383, *p* < 0.001). However, the wide CI observed in the model limits the reliability of this result. Therefore, this finding should be interpreted with caution and considered as a potential trend rather than conclusive evidence of a strong association. Previous studies have reported inconsistent results regarding the relationship between rs17817449 and insulin resistance across different populations [34, 35]. The limited sample size or environmental factors in our study may have contributed to this discrepancy. These results may lead to the development of new strategies for diabetes prevention and management of complications in Turkish children with FTO gene polymorphism.

Some studies have shown an association between rs9939609 polymorphism and insulin resistance. HOMA‐IR values were found to be higher in the AA genotype in Chilean [38], Spanish [39], and Polish [40] children. In a study conducted with Iraqi patients with obesity, rs9939609 and rs17817449 polymorphisms were shown to increase insulin resistance and thus increase the risk of Type 2 diabetes. At the same time, the GT genotype of the rs17817449 polymorphism has been reported to increase the risk of Type 2 diabetes [41]. Similar to the results of our study, it has been reported that there is an association between rs17817449 polymorphism and insulin resistance, and children with GT genotype have higher HOMA‐IR values [42]. However, in Iran, no association was found between rs17817449 polymorphism and insulin resistance [43]. In light of these results, it can be concluded that the genotypes of the FTO gene rs17817449 and rs9939609 polymorphisms are specifically more prone to insulin resistance and that this may change depending on the ethnic origin. In future studies, current gene polymorphisms should be investigated to prevent childhood obesity and related cardiometabolic disorders.

In childhood obesity, liver enzymes, especially ALT level may be increased due to non‐alcoholic fatty liver disease. In a study conducted in children with obesity, ALT and ALT/AST ratio were associated with blood lipids and insulin resistance [44]. It has also been stated that elevation of liver enzymes in the presence of insulin resistance may be an indicator of non‐alcoholic fatty liver [45]. Similar to the literature [46], in our study, it was determined that children with the rs9939609 polymorphism risk genotype (AA) had a higher ALT/AST ratio. On the other hand, this rate was found to be lower in children with the risk genotype (GG) for the rs17817449 polymorphism (*p* < 0.05). In this context, it can be said that FTO gene polymorphisms cause different effects on liver enzyme levels [47]. Therefore, polymorphisms effective in the synthesis of liver enzymes should be investigated more comprehensively and current methods should be introduced to prevent obesity‐related complications.

There are some limitations to our study for various reasons. Firstly, the number of children included in the study is less than in other studies conducted on a large population due to the low number of patients admitted to the hospital. It may be beneficial to conduct studies to evaluate the effect of FTO polymorphisms on insulin resistance in Turkish children with obesity using larger samples. Secondly, since the aim of the study was to evaluate the relationship between FTO gene rs9939609 and rs17817449 polymorphisms and insulin resistance in children with obesity, children with obesity without insulin resistance were included in the study instead of healthy children as a control group. Finally, the effects of FTO gene rs17817449 and rs9939609 polymorphisms, which are associated with insulin metabolism, on body composition were not evaluated in our study. Studies to investigate the effect of FTO gene polymorphisms on different body compositions can be planned.

## 5 Conclusion

As a result, a strong relationship was found between FTO gene rs17817449 (GT genotype) and rs9939609 (AA and AT genotypes) polymorphisms and insulin resistance, regardless of age, gender, and BMI. Additionally, fasting glucose, insulin, and QUICKI values differed between the two polymorphisms. These results may provide new perspectives on improving insulin resistance and preventing Type 2 diabetes in Turkish children with FTO gene polymorphism. It is thought that the effects of different gene polymorphisms on insulin metabolism, biochemical parameters, chronic diseases, and body composition should be investigated more comprehensively and longitudinally in future studies.

## Author Contributions

Merve Köksal and Mehtap Ünlü Söğüt conceived and designed the study. Sevtap Kabalı contributed to the design. Merve Köksal and Mehtap Ünlü Söğüt conducted the study. Sevtap Kabalı did the statistical analysis. Merve Köksal, Mehtap Ünlü Söğüt, and Sevtap Kabalı interpreted the data. Merve Köksal, Mehtap Ünlü Söğüt, and Sevtap Kabalı drafted the original manuscript. All authors reviewed and revised the manuscript.

## Funding

This work was supported by Ondokuz Mayis Üniversitesi, OMU‐BAP, Project No: PYO.SBF.1904.18.009.

## Ethics Statement

The necessary permissions were obtained from the Ministry of Health of the Republic of Türkiye for the study (Decision no: TUEK1‐2019BADK/1‐4, Date: 08.01.2019). The study was approved by the Clinical Research Ethics Committee, Ondokuz Mayıs University, Samsun, Türkiye (Decision no: 2018/269, Date: 25.06.2018) and all procedures were performed in accordance with the principles of the Declaration of Helsinki.

## Consent

The parents and children were informed by the researchers about the purpose of the study and written informed consent was obtained from each of them.

## Conflicts of Interest

The authors declare no conflicts of interest.

## Data Availability

The data that support the findings of this study are available from the corresponding author upon reasonable request.
